# The Preparation and Characterization of Polyacrylonitrile-Polyaniline (PAN/PANI) Fibers

**DOI:** 10.3390/ma12040664

**Published:** 2019-02-22

**Authors:** Iwona Karbownik, Olga Rac-Rumijowska, Marta Fiedot-Toboła, Tomasz Rybicki, Helena Teterycz

**Affiliations:** 1 Faculty of Electrical, Electronic, Computer and Control Engineering, Technical University of Łódź, Żeromskiego 116, 90-924 Łódź, Poland; ivakabari@gmail.com (I.K.); trybicki@p.lodz.pl (T.R.); 2 Faculty of Microsystem Electronics and Photonics, Wrocław University of Science and Technology, Janiszewskiego 11/17, 50-372 Wroclaw, Poland; helena.teterycz@pwr.wroc.pl; 3 Polish Centre for Technology Development PORT, Stabłowicka 147, 54-066 Wroclaw, Poland; marta.fiedot-tobola@eitplus.pl

**Keywords:** polyacrylonitrile, polyaniline, conductive fibers

## Abstract

The paper presents a method of modifying polyacrylonitrile (PAN) fibers using polyaniline (PANI). The PAN fibers were doped with polyaniline that was obtained in two different ways. The first consisted of doping a spinning solution with polyaniline that was synthesized in an aqueous solution (PAN/PANI blended), and the second involved the synthesis of polyaniline directly in the spinning solution (PAN/PANI in situ). The obtained fibers were characterized by the methods: X-ray powder diffraction (XRD), scanning electron microscope (SEM), fourier-transform infrared spectroscopy (FTIR), thermogravimetry (TG) and differential scanning calorimetry (DSC). Analysis of the results showed strong interactions between the nitrile groups of polyacrylonitrile and polyaniline in the PAN/PANI in situ fibers. The results of mechanical strength tests indicated that the performance of the PAN/PANI mixture significantly improved the mechanical parameters of polyaniline, although these fibers had a weaker strength than the unmodified PAN fibers. The fibers obtained as a result of the addition of PANI to PAN were dielectric, whereas the PANI-synthesized in situ were characterized by a mass-specific resistance of 5.47 kΩg/cm^2^.

## 1. Introduction

Fibers, regardless of whether they are a natural or synthetic product, are used primarily for the production of clothing and technical fabrics. Recently, more and more attention has been paid to giving completely new properties to fabrics, such as antibacterial or photocatalytic properties, as well as properties of electrical conductivity. Electrically conductive fibers can be used in textronic products, which are multifunctional textile materials that have the features of standard textile products, but at the same time have the functions of electronic components. Electroconductive fibers, depending on their resistance, can be used as electrical connections, sensors, or electromagnetic field shielding materials. However, the mechanical properties of the electrically conductive fibers must allow their further processing in order to obtain a textile product.

Polyaniline, apart from polypyrrole (PPy) or poly(3,4-ethylenedioxythiophene) (PEDOT), is one of the most popular conductive polymers because of its reversible redox, pH-switching, and sensing properties, and its simple synthesis [1]. However, like most of these types of materials, it has poor mechanical properties and thermal stability. Due to the fact that it is difficult to use melt techniques like extrusion, polyaniline fibers are formed only from spinning solution in a spinning process [2]. The polyaniline fibers were initially obtained from pure polyaniline, and in order to increase its electrical conductivity it was doped with concentrated sulfuric acid during the preparation of the spinning solution. This improves the electrical conductivity, but weakens the mechanical properties of the fibers [3]. One of the solutions is to use high molecular weight polyaniline [4,5]. However, the most common solution is to combine polyaniline with other polymers to increase its mechanical strength and to facilitate its processing. For this purpose, polymers known for their high strength parameters, poly–ω-aminoundecanoyle (nylon-11) and poly-phenylene terephthalamide (Kevlar), were used in a wet-spinning process [6,7,8,9]. These fibers were characterized by a much higher mechanical strength in comparison to pure polyaniline fibers; however, they had several orders of magnitude lower electrical conductivity [2].

One of the polymers used for this purpose is also a polyacrylonitrile, which is characterized by high mechanical strength, as well as thermal and chemical resistance, and also low price. In addition, it is a fiber-forming polymer popularly used for the production of carbon fibers. 

Zhai et al. obtained flexible and conductive layers as a result of direct polymerization of aniline on the surface of microporous polyacrylonitrile. The use of PAN improved the mechanical properties and thermal stabilities of the polyaniline layers [10]. The combination of PAN and PANI polymers is used in medicine, where copolymer is used as scaffolds for muscle cells [11]. Polymer membranes were made from the PANI-doped polyacrylonitrile, and their color depended on the pH of the solution [12]. Moreover, fibers from the PAN/PANI composition were made by electrospinning, where an increase in electrical conductivity towards polyacrylonitrile was observed [13]. Toptas et al. obtained conductive polyaniline/polyacrylonitrile composite fibers by sorption of aniline and polyacrylonitrile fiber [14]. These fibers had satisfactory electrical conductivities, mechanical strengths, and resistances for laundering. Xia and Lu obtained conductive fibers in which polyacrylonitrile fibers were coated with polypyrrole, polyaniline, and poly (3,4-ethylenedioxythiophene). The polymerization occurred directly on the surface of the PAN fibers, by way of a chemical connection between polyacrylonitrile chains and conjugated polymers that was created [15]. There are also studies in which polyaniline fibers are doped with carbon nanotubes (CNT) with high electrical conductivity and high mechanical strength [16].

This article presents the results of research on PAN/PANI fibers, obtained using the wet method, from solutions in which the polymer matrix was polyacrylonitrile (PAN) and admixed polyaniline (PANI). PANI was introduced to PAN fibers using two methods:

- the blending method, where PANI was mixed with the spinning solution,
- in situ synthesis of polyaniline in a spinning solution.

The present method, where polyaniline is synthesized in situ during the preparation of a spinning solution for forming PAN/PANI composite fibers by wet spinning, is new and has not been described in literature.

## 2. Materials and Methods 

### 2.1. Materials

As a fiber-forming polymer, polyacrylonitrile (PAN) (Good-Fellow Co., Huntingdon, UK) was used, which contained 99.5 wt.%. polyacrylonitrile and 0.5 wt.%. methyl polyacrylate. Polyaniline was prepared from aniline chloride C_6_H_7_N·HCl (Sigma-Aldrich Co., St. Louis, MO, USA) and ammonium persulphate (NH_4_)_2_S_2_O_8_ (POCH, Gliwice, Poland). Dimethylformamide (DMF) (Chempur, Piekary Śląskie, Poland) was used as a solvent for the polyacrylonitrile.

### 2.2. Blended Polyaniline (PANI) with Spinning Dope

Polyaniline (PANI) was obtained by mixing an aqueous solution of 0.5 M aniline hydrochloride (C_6_H_7_N·HCl) and 0.6 M ammonium persulfate ((NH_4_)_2_S_2_O_8_). Ammonia was added to the mixture after 4 h and the resulting precipitate was filtered using a Schott funnel. The obtained precipitate was washed with methanol, dried at room temperature, and then ground to a fine powder. The polyaniline powder was dissolved in dimethylformamide under the influence of ultrasound. Polyacrylonitrile (PAN) was added to the obtained solution. The weight concentration of polyacrylonitrile in the spinning solution was 13.5%, and polyaniline was approximately 1 wt.% to the weight of polyacrylonitrile. The fibers obtained using this method were marked as PAN/PANI blended.

### 2.3. Synthesis of PANI in Spinning Dope

**Figure 1:**
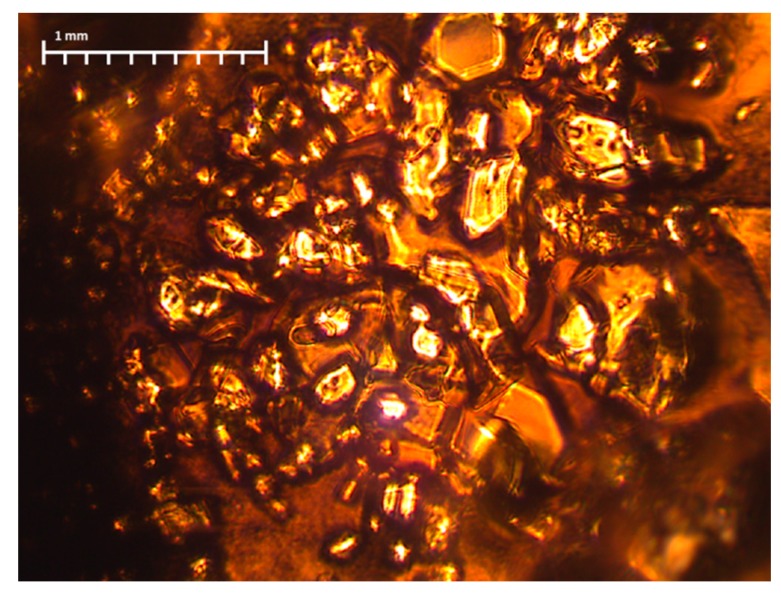
Crystals precipitated during the reaction of polyaniline in dimethylformamide (DMF) after filtration.

The literature data shows that the best convertible polyaniline is obtained in the solution polymerization process [17]. For this reason, in the second method, polyaniline was synthesized in situ during the preparation of the polyacrylonitrile spinning solution in dimethylformamide (DMF). Two solutions in dimethylformamide (containing 1% of dissolved PAN) were prepared with 0.5 M aniline hydrochloride and 0.6 M ammonium persulfate. Both solutions were mixed together for 4 h. The reaction was carried out at a temperature not exceeding 4 °C. After 20 h, the obtained reaction mixture was filtered because colorless crystals were precipitated during the reaction (Figure 1). 

Polyacrylonitrile was added to the filtered solution. The solution was heated to 40 °C and stirred for 4 h, then cooled for 2 h. In the synthesis of the polyaniline, the same amount of substrates as in the blended method was used. The fibers obtained using this method were marked as PAN/PANI in situ.

### 2.4. Spinning of Fibers

**Figure 2:**
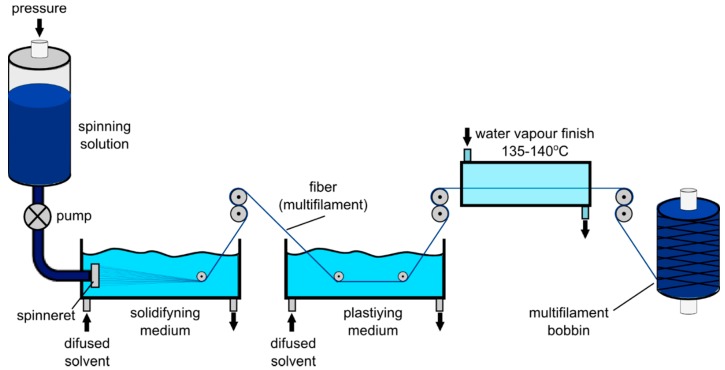
Scheme of fiber spinning using the wet method.

Polyacrylonitrile (PAN) fibers and both types of polyacrylonitrile-polyaniline fibers (PAN/PANI) were obtained using the wet method. A reference fiber spinning solution (PAN) was prepared by dissolving polyacrylonitrile (13.5%) in dimethylformamide (DMF) at 40 °C. After passing through the nozzle, the spinning solution was introduced into a solidifying bath, in which PAN coagulated in the form of fibers (Figure 2). In a solidifying bath (60% aqueous DMF solution) at 20 °C, and in a plasticizing bath (50% aqueous solution of DMF) at a temperature of 70 °C, intensive removal of the solvent and impurities from the formed fibers took place. The fibers underwent a two-stage stretching process—in the plasticizing bath and in water vapor at 135 °C. During these processes, the orientation of macromolecules took place and the fiber structure was ordered, which improved the mechanical properties of the fibers. The PAN/PANI in situ fibers were not subjected to stretching in water vapor because of tearing during the tensile test. The formed and stretched fibers were wound on reels. 

### 2.5. Characterization

The molecular weights of polymers were determined by gel permeation chromatography (GPS) using dimethylacetamide (DMAc) as a solvent with the addition of 0.5 wt.% lithium chloride (LiCl). Rheological measurements of the spinning solutions were obtained by a ReolabQC Anton Paar rheoviscometer (Anton Paar, Ostfildern, Germany). The kinetics of polyaniline formation in the DMF were investigated using UV-Vis spectroscopy with a UV-Vis-NIR JASCO V-570PC spectrophotometer (Jasco, Easton, MD, USA). Photographs of the fibers were made using a Leica DM 4000M Led microscope (Leica, Wetzlar, Niemcy). The chemical composition was determined on the basis of infrared spectroscopy with a Nicolet 380 FTIR spectrophotometer (Thermo Fisher Scientific, Waltham, MA, USA) with an ATR Smart Performer (Thermo Fisher Scientific, Waltham, MA, USA). A PC-controlled low-load machine was used for testing the mechanical properties of the fibers formed (Zwick Z2.5/TN1S, Ulm, Germany), according to the PN-EN ISO 2062:2010 standard [18]. The fiber samples’ morphology was analyzed with a TESCAN VEGA3–EasyProbe (TESCAN, Brno, Czech Republic), and a scanning electron microscope (TESCAN, Brno, Czech Republic) equipped with VEGA TG software (TESCAN, Brno, Czech Republic) was used for morphological analysis of the doped polyacrylonitrile fibers (high vacuum mode (SE); accelerating voltage 7–20 kV). Before the measurements, the samples were sputtered with Au-Pd (SC7620 Mini, Quorum Technologies Ltd., Lewes, UK) for 120 s. The cross-section of the fibers was observed and obtained using a high-resolution SEM Xe-PFIB FEI Helios (Brucker, Billerica, MA, USA). A single fiber was covered with a platinum layer, and then a cross-section was made with a focus ion beam (FIB). Thermal gravimetric analysis (TGA) was carried out using a thermobalance TGA Mettler Toledo (Columbus, OH, USA) in the temperature range from 25 to 600 °C and a heating speed of 10 °C/min. A corundum crucible with a hole in its lid of 70 μL was used. Measurements were carried out under nitrogen, and the flow rate was 30 mL/min. Differential scanning calorimetry (DSC) measurements were performed using a DSC Mettler Toledo (Columbus, OH, USA) in the temperature range from 25 to 200 °C with a heating/cooling rate of 10 °C/min. Measurements were carried out under nitrogen, and the flow rate was 30 mL/min in a corundum crucible with one hole in its lid of 40 μL. The study of electrical conductance was investigated using the direct current (DC) method with the use of a Keithley 2000 multimeter (Cleveland, OH, USA). Measurements were made on 1 cm lengths between two silver electrodes. 

## 3. Results and Discussion

### 3.1. Molecular Weight of Polymers

Based on the results of exclusion chromatography (GPC) analysis, the molecular weights of the polymers were determined. The average molecular weight of polyacrylonitrile was Mw 644,600 and Mn 177,400. Its polydispersity was 3.6, which indicates a very large weight distribution of molecules in the polymer. On the other hand, the average molecular weight of the PANI obtained in the aqueous solution, which was Mw 12,920 and Mn 4263, respectively, and the degree of polydispersity of PANI were also large, amounting to 3.0.

### 3.2. Rheological Properties of Spinning Solutions

The basic properties of each spinning solution, which demonstrated its quality, were rheological properties. To modify the composition of these solutions, it was necessary to determine the effect of modifications on these properties. When analyzing the rheological properties of spinning solutions, the dependence of tangential stress on shear rate (A power-law fluid—the Ostwald–de Waele relationship) is analyzed (1):

(1) $$\tau =k\gamma^{n}$$

where n is the flow behavior index (-), and k is the flow consistency index (Pa·s^n^). These two basic rheological parameters of each spinning solution were determined experimentally (Table 1).

The value of the flow behavior index depended on the way in which polymer macromolecules were placed in motion, and the way they formed a system of mutually sliding layers. The linear polymer chains in the spinning solution (with a sufficiently high concentration) can be highly tangled. The determined value of n (Table 1) indicated that all the obtained spinning solutions were shear thinned liquids. This is desirable for technological reasons (the ease of pressing the solution through the nozzles). The flow rates of solutions containing polyaniline were slightly higher. This means that polyaniline particles minimally affected the orientation of polyacrylonitrile macromolecules. This gentle effect on the *n*-value may be due to the difference in the molecular structure of both polymers. The flow behavior index depended on several factors, e.g., the average molecular weight of the polymer and its degree of polydispersity, the degree of branching of macromolecules, and the content of additional components (especially fillers) and plasticizers. In the studied system, both polymers were linear, but the PAN macromolecules formed entangled structures, and the PANI macromolecules were largely rigid due to the presence of quinone rings in them. This parameter, in addition to temperature, pressure, and concentration, had a significant impact on the value of the flow consistency index k. Taking into account technological requirements, the k value of spinning solutions should be greater than 20 Pa·s^n^. In comparison to changes in the n value, the presence of polyaniline molecules in the spinning solution caused a significant change in the k value. This change was mainly due to the addition of a polymer with a much lower average molecular weight relative to the spinning solution. The average molecular weight of PANI was 50 times less than PAN. Moreover, a significantly lower value of the consistency coefficient of spinning solutions containing PANI may be related to the occurrence of interactions between the PAN and PANI chains [19,20].

### 3.3. Fiber Morphology

It is well known that polyaniline obtained by aniline polymerization can occur in three oxidation states [21]:

- leucoemeraldine—(C_6_H_4_NH)_n_—completely reduced, white or colorless;
- emeraldine—([C_6_H_4_NH]_2_[C_6_H_4_N]_2_)_n_—blue for the emeraldine base, green for the emeraldine salt;
- (per)nigraniline—(C_6_H_4_N)_n_—completely oxidized, blue or violet.

Of all forms of polyaniline, only emeraldine salt (green) has an electrical conductivity at the level of semiconductors, i.e., around 1 S/cm. Emeraldine salt is obtained as a result of emeraldine base modification by HCl or HBr. Each type of polyaniline can be transformed in a reversible process of oxidation or reduction, during which changes in color and electrical conductivity occur [22].

**Figure 3:**
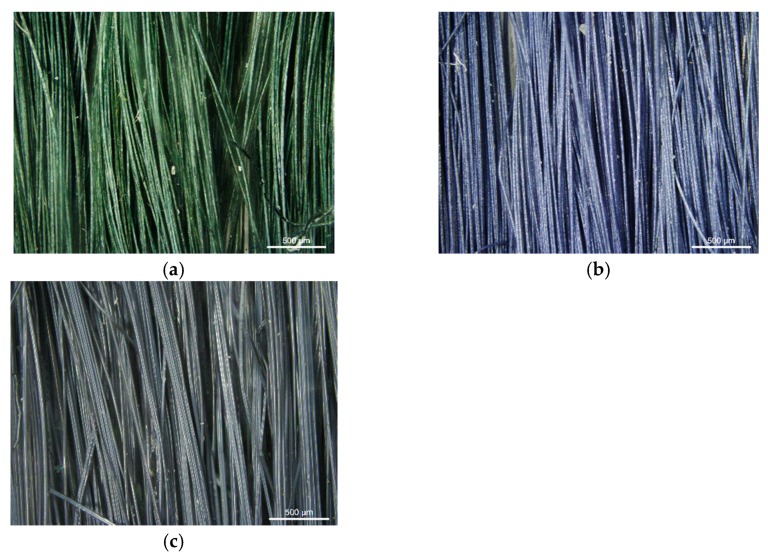
Fiber images made with an optical microscope. Fibers obtained as a result of the addition of polyaniline (PANI): (**a**) after rinsing in water; (**b**) after rinsing in HCl; and (**c**) fibers obtained as a result of in situ synthesis of PANI.

The PAN/PANI blended fibers obtained by adding polyaniline powder to the spinning solution had a dark green color (Figure 3a), which, after immersing them in a 3% aqueous solution of hydrochloric acid, turned to dark blue (Figure 3b). After rinsing again in water, their color returned to the previous color. The fibers obtained as a result of the synthesis of polyaniline in situ in the spinning solution were dark navy (Figure 3c).

Based on the color of the obtained fibers, it can be concluded that polyaniline in the PAN/PANI blended fibers was in an emeraldine base form, and in an HCl solution it transformed into emeraldine salt (Figure 3b). Moreover, in the PAN/PANI in situ fibers, polyaniline was in the emeraldine base form.

(Per)nigraniline—(C_6_H_4_N)_n_—a completely oxidized form of PANI—also has a blue or violet color. However, taking into account that the synthesis of polyaniline was carried out in a solvent (DMF), which is a strong reducer, it cannot be assumed that a fully oxidized form of PANI was formed. In addition, during the synthesis of PANI in DMF, colorless crystals were formed, which indicated that a part of polyaniline precipitated in the form of leucoemeraldine—a completely reduced form. A significant part of the leucoemeraldine was filtered during the synthesis, and, therefore, the fibers were mostly polyaniline fractions with shorter chains.

### 3.4. UV–Vis Spectroscopy

**Figure 4:**
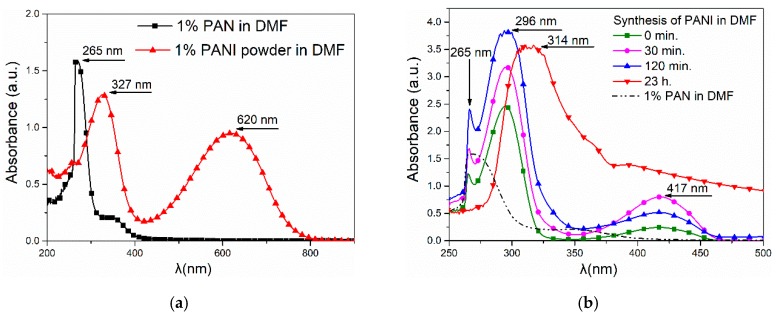
UV-Vis spectrum: (**a**) polyacrylonitrile (PAN) and PANI powder dissolved in Dimethylformamide (DMF) (blended); (**b**) change in absorbance during the PANI synthesis in DMF (in situ).

On the UV-Vis spectrum of PANI solution in DMF, two peaks were visible (Figure 4). The peak at 323 nm was connected with π-π* excitation, and the peak at 618 nm was connected with the exciton excitation of the quinone rings in the PANI (Figure 4a). The presence of these peaks and the dark blue coloration of the solution were characteristic for the emeraldine base [23]. On the spectrum of the spinning solution containing 1 wt.% of polyacrylonitrile and polyaniline synthesized in DMF, the peak near 320 nm was more visible and shifted in towards the shorter wave (Figure 4b). However, in this spectrum there was no clear peak around 618 nm, which corresponded to the exciton excitation of the quinone rings in the PANI. 

The PANI synthesis in the DMF solution containing 1 wt.% of PAN occurred very slowly. In this time, the peak characteristic for PAN disappeared. In contrast, the characteristic peak, corresponding to the π-π* excitation during synthesis, moved towards the 327 nm band. This can indicate the interaction between PAN and PANI molecules [24].

### 3.5. FTIR Spectroscopy

**Figure 5:**
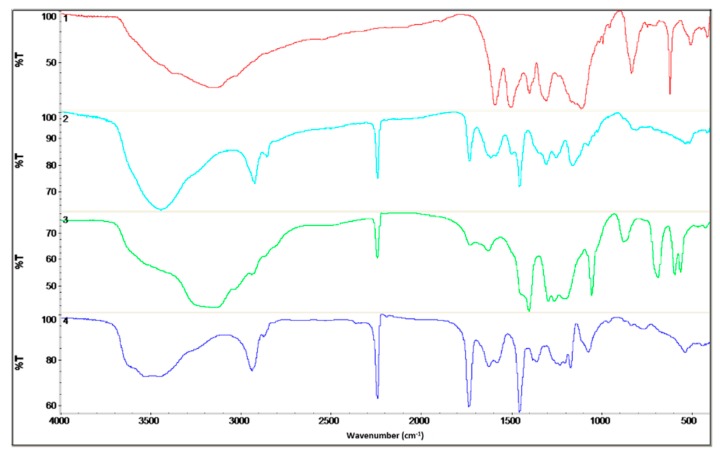
FTIR Spectra from the top: PANI powder (red); fibers: PAN/PANI blended (cyan); PAN/PANI in situ (green); and PAN (blue).

The chemical structure of the polyaniline, polyacrylonitrile, and obtained fibers was determined on the basis of infrared spectroscopy (Figure 5). A description of the characteristic vibrations is shown in Table 2 [15,25].

On the fibers’ FTIR spectra, the bands characteristic of polyaniline and polyacrylonitrile were visible. In the case of PAN/PANI blended fibers, all absorption bands characteristic of polyacrylonitrile were clearly visible.

In the case of PAN/PANI blended fibers, all absorption bands characteristic of polyacrylonitrile were clearly visible at 3445, 2926, 2243, 1731, 1615, 1453 and 1250 cm^−1^. The peaks corresponded to N–H stretching and C–H stretching in the polymer structure, stretching in C≡N, stretching in C=O, stretching in C=C, bending C–H in CH_2_, and stretching in C–N. The gentle absorption bands characteristic of PANI were also visible at 1588, 1160 and 804 cm^−1^, which were characteristic of C=C stretching in the quinoid rings, C–N bond stretching in quinoid and benzenoid rings, and δ C–H in 1,2,4 trisubstituted benzene rings. FTIR analysis indicated that these fibers were composed of polyacrylonitrile and had a low concentration of polyaniline incorporated in them, as evidenced by the low intensity of the bands characteristic of this polymer.

On the other hand, in the PAN/PANI in situ fiber spectrum, the absorption bands associated with the presence of PANI-specific bonds were much more intense. A fuzzy peak above 3000 cm^−1^ was also visible, which was associated with the presence of water. This is not present in the PAN/PANI blended spectrum. The bands occurring at 1414, 1194 and 879 cm^−1^ were characteristic of stretching in C–N between benzene and quinoid rings, and bending in C–H and 1,2,4 trisubstituted benzene rings. The absorption bands characteristic for polyacrylonitrile were also visible at 2940, 2244, 1726, 1629, 1445 and 1260 cm^−1^, which was characteristic of C–H stretching in polymer structures, stretching in C≡N, stretching in C=O, stretching in C=C, bending C–H in CH_2_, and stretching in C–N.

In addition, a band corresponding to the vibrations of the deprotonated polyaniline form (1160 cm^−1^) was present in both fibers containing polyaniline. Moreover, pure polyaniline was in a protonated form. 

From the shifts in absorption bands in the PAN/PANI in situ fiber spectra, relative to unmodified polyacrylonitrile and polyaniline, it can be concluded that electrostatic interactions arose between the chains of these polymers between the polyaniline and nitrile groups. The confirmation of the interaction between polyaniline and the nitrile group of polyacrylonitrile was in the reduction of the intensity of the absorption band around 2244 cm^−1^ associated with stretching vibrations in C–N, and also the band around 3480 cm^−1^ associated with stretching N–H vibrations. In both cases, this reduction was more visible in the PAN/PANI in situ fibers. Such interactions between different conductive polymers and polyacrylonitrile are known in the literature [15]. These results confirmed observations drawn from the research of the rheological properties of the obtained solutions and UV-Vis spectra.

### 3.6. SEM Images

**Figure 6:**
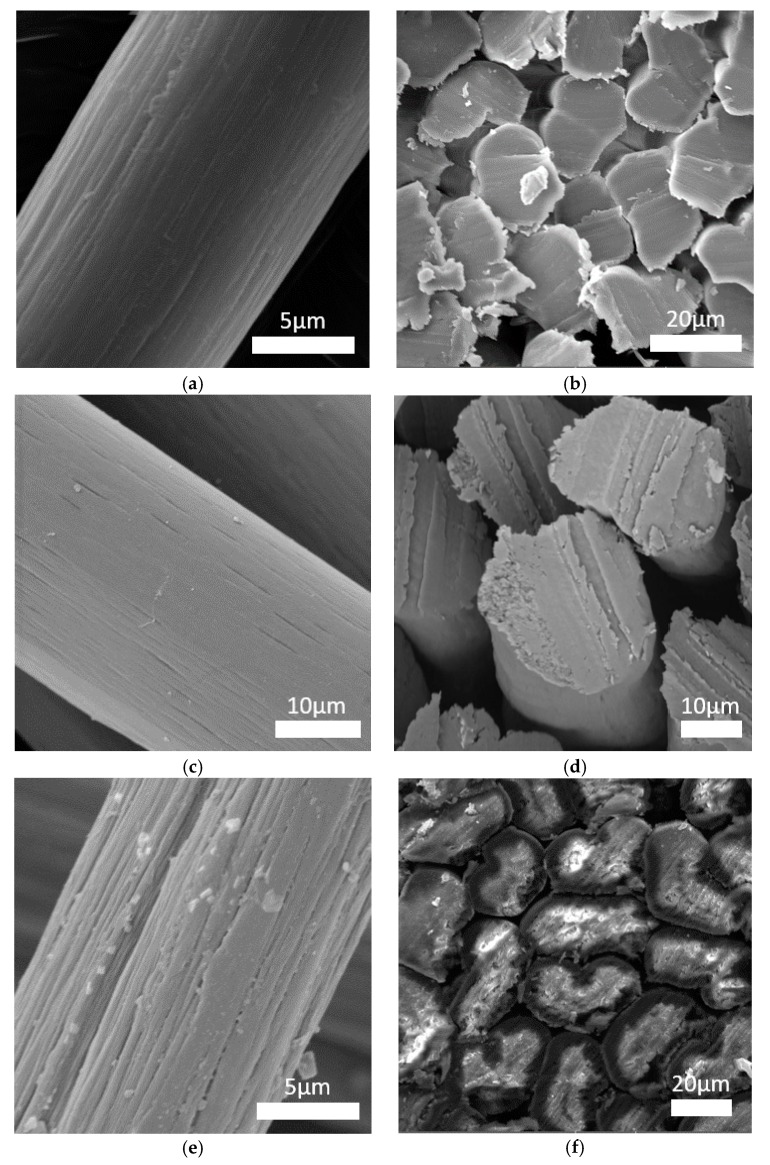
SEM images of fibers: (**a**,**b**) PAN; (**c**,**d**) PAN/PANI blended; and (**e**,**f**) PAN/PANI in situ.

**Figure 7:**
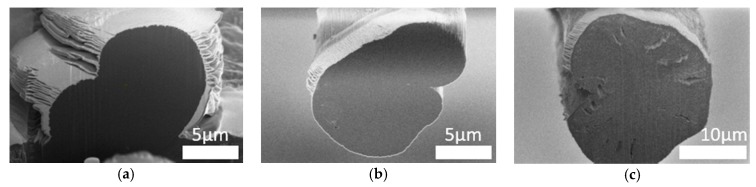
SEM images of the cross-section of: (**a**) PAN; (**b**) PAN/PANI blended; and (**c**) PAN/PANI in situ.

The microstructure of the fibers was observed using a scanning electron microscope (Figure 6). Cross-sections of individual fibers were also made using a focus ion beam (FIB) microscope (Figure 7). The surface morphology and fiber cross-sections were different for every fiber. The standard PAN fibers had a visible fibrous structure with characteristic furrows on their surface (Figure 6a). The cross-sections of these fibers had a characteristic bean-like shape (Figure 6b). The surface of the PAN/PANI blended fibers treated with hydrochloric acid was very smooth, and their cross-section was oval (Figure 6c,d and Figure 7b). Deep furrows were clearly visible on the surface of the PAN/PANI in situ fibers. The cross-sections of these fibers showed pores and cracks (Figure 7c). The cross-section shape was also bean-like, but the surface structure was clearly different from the inside of the fibers (Figure 6e,f). The structure of the PAN/PANI in situ fibers was discontinuous and contained many defects. It was clearly more heterogeneous than in the PAN/PANI blended fibers (Figure 6e,f). 

The reason for such differences in the microstructure of fibers can be, above all, the difference in the size of the macromolecules that have been in the solution (see Section 3.1), and also the amount of tension during the spinning process (see Section 2.4). As previously mentioned, during the synthesis of the PANI in the DMF and PAN solution, transparent crystals were formed (Figure 1), which were then filtered off. Thus, only low molecular weight molecules were left in the solution, which hindered the fiber formation process. Moreover, according to FTIR results (Figure 5) (Table 2), during the synthesis of PANI in the DMF and PAN solution, new interactions appeared between the functional groups of both polymers. This could weaken the interaction between the polyacrylonitrile chains themselves.

### 3.7. Mechanical Strength

Polyaniline is a highly used material, primarily due to its high electrical conductivity, low price, and its easy method of obtaining. However, the biggest difficulty associated with its use is its low mechanical strength and processing problems. For this reason, the fibers were made from a mixture of polyaniline with fiber-forming polyacrylonitrile, which, in this system, was a polymer responsible for mechanical strength.

The fiber linear mass was determined using the following Equation (2):

(2) $$m_{l}=\frac{m}{l}\times 1000\left[\frac{g}{m}\times 1000=\mathrm{tex}\right],$$

where m is the average sample weight and l is the length of the sample. 

The linear mass of the PAN/PANI blended fibers was slightly higher than the PAN fibers (Table 3). However, the linear mass of the PAN/PANI in situ fibers was more than twice as high. This was because the fibers were thick as a result of them both being subjected to stretching in steam.

Based on the breaking curve, the most important parameters of the mechanical strength of the fibers were determined (Table 3). The Young’s modulus and the specific strength of both polyaniline-containing fibers was lower than that of the pure polyacrylonitrile fibers, with the reduction being more pronounced in the case of the fibers modified in situ (Table 3). 

During the stretching of fiber-forming polymers, their crystalline structure is shaped, which increases the mechanical strength of the fibers. However, as previously mentioned, the PAN/PANI in situ fibers were not subjected to stretching in steam. While stretching, the fibers were easier to delaminate and less likely to break. This effect may occur when adding an incompatible polymer or when adding a polymer that changes the interactions between the fiber-forming polymer chains. Both the PANI/PANI blended and PAN/PANI in situ fibers contained the same polymer, and, therefore, the delamination effect was difficult to explain by the presence of an incompatible polymer.

However, on the basis of the FTIR results (Figure 5), interactions between these polymers were found, which probably caused a reduction in the interactions between the PAN macromolecules, as well as a significant reduction in the consistency coefficient (Table 1). In addition, if it was assumed that a small molecule polyaniline remained in the polyacrylonitrile solution during the fiber coagulation process, it would pass into the coagulation bath, causing pores to be visible on SEM images (Figure 6f,c).

Typically, thermoplastic polymers such as Nylon or Kevlar [7,8] are used as PANI strength-enhancing materials. However, the presence of these polymers causes a decrease in the electrical conductivity of the PANI. This inversely proportional relationship is associated with the presence of micropores in the fibers, which on the one hand reduces the mechanical strength, but on the other hand facilitates the penetration of an admixture, such as HCl, into the structure of a fiber. This penetration, in turn, increases the conductivity of the fibers [26,27]. For this reason, the generation of electrically conductive fibers based on electronically conductive polymers has many denominations, of which, the greatest seems to be the achieving of both the desired mechanical properties and a satisfactory electrical conductivity.

### 3.8. XRD

**Figure 8:**
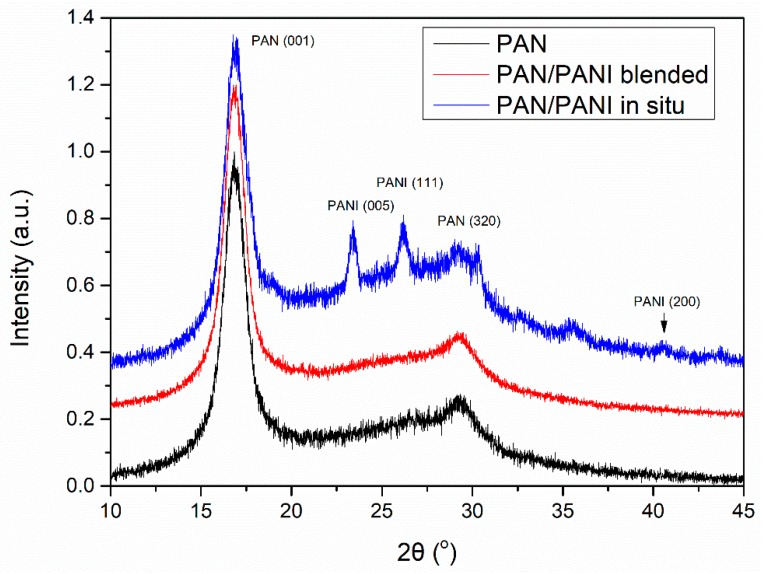
Diffractogram of the fibers.

The fiber crystal structure was determined based on XRD studies (Figure 8). On all diffractograms, peaks characteristic of polyacrylonitrile were visible. Particularly clear was the peak at 2θ~18°, which was characteristic of the hexagonal structure of PAN. In the PAN/PANI blended fiber spectrum there were no peaks characteristic of PANI, most probably due to its small amount in the fibers. However, they were clearly visible in the PAN/PANI in situ fibers, and corresponded to the pseudo-orthorhombic phase of PANI [28].

The degree of fiber crystallinity (X_c_) was calculated using the Hinrichen method (3):

(3) $$X_{c}=\frac{I_{c}}{I_{c}+I_{a}},$$

where I_c_ is the integral under the peaks corresponding to the crystalline phase of the polymer, and I_a_ is the integral under the peaks corresponding to the amorphous phase of the polymer. 

Crystal and amorphic peaks were determined based on the optimization carried out in the WAXFIT program. Curve fitting was performed using the Rosenbrock method. The degree of crystallinity for the PAN and PAN/PANI blended fibers was the same at 0.53, while for the PAN/PANI in situ fibers it was significantly lower at 0.32 (Table 4). The significantly lower crystallinity degree of the PAN/PANI in situ fibers was caused by a lack of stretching, and was correlated with their mechanical strength. 

The higher degree of crystallinity, in the case of fibers, was associated primarily with a larger ordering of polymer chains along the fiber axis [29]. This parameter depended on the type and degree of interactions between macromolecules.

**Figure 9:**
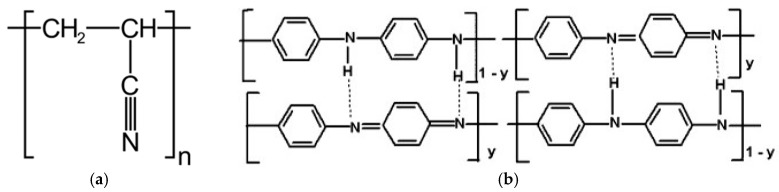
Chemical structure of: (**a**) PAN; and (**b**) PANI.

The polyacrylonitrile macromolecules contained positively charged CN groups (Figure 9a), and caused the chains to twist into a helix, from which the crystallites are made [30]. In the PANI, due to the presence of amine groups, hydrogen bonds and dipole-dipole interactions were formed between the macromolecules (Figure 9b). H-bonding interactions between adjacent chains caused the stiffness of the PANI chain [31], and an easier orientation of the chains in the material containing only polyaniline.

**Figure 10:**
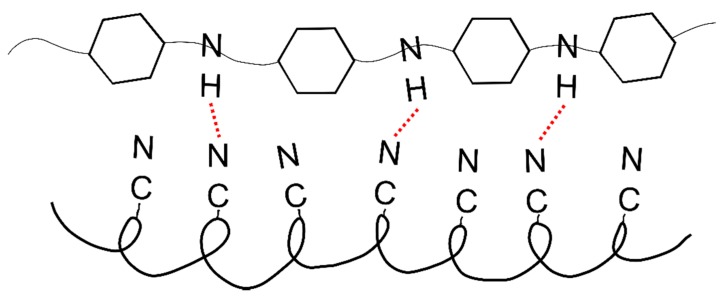
Diagram of interaction between the PAN and PANI chains.

However, in the material containing both of these polymers, the crystallization process was difficult due to the interactions between nitrile (PAN) and amine (PANI) groups (Figure 10). In addition, numerous cracks and voids appeared in the structure of the fiber (Figure 6c). Analysis of the literature data [32] indicated that the nitrile group, in some cases, may have interfered with or acted as a barrier when ordering polymer chains. This can take place in the PAN/PANI in situ fibers, where the strong interaction between the polymer chains blocks their movement, thereby limiting the crystallization process.

The average size of PAN crystallites was determined based on the half-width of the peak 2θ~17°, characteristic of the plane (001) in a hexagonal structured PAN. The size of the PANI crystallites was determined based on the peaks 2θ~23° and 26°, characteristic of the planes (005) and (111). The calculations were made using the Debye–Scherrer Equation (4):

(4) $$L_{\mathrm{hlk}}=\frac{K\times \lambda }{\beta \times {\cos\theta }_{\max}},$$

where L_(hlk)_ is the average size of crystallites, K is the Scherrer constants (0.89), θ_max_ is the angle for the maximum peak (rad), λ is the length of the radiation beam (Å), and β is the half-width of the peak (rad).

The inter-chain separation length (R) was determined based on the analysis of the most intense crystalline peak from the following Equation (5) [31]:

(5) $$R=\;\frac{5\lambda }{8\sin\theta_{\max}}.$$

The distance between planes (the d-spacing) was determined from the Bragg Equation (6):

(6) nλ=2dsinθ,

where n is the deflection integer.

Lattice strain ε was determined from Formula (7):

(7) β=4εtanθ

Data on the crystal structure of the obtained fibers was collected in Table 5. The crystal phase of the polyacrylonitrile in all the fibers had a similar structure. Crystallite size, d-spacing, inter-chain separation, and lattice strain all had very similar values. The size of the polyaniline crystallites was larger and amounted to 15.8 (111) and 17.9 (005) nm. Differences in the structure of both chemical and crystalline polyacrylonitrile and polyaniline present in fibers were reflected in their macroscopic structure and mechanical properties.

Inter-chain separation length is a parameter affecting the electrical conductivity of fibers. This value represents the distance between the electron jump between the chains. The smaller it is, the higher the probability of charge hopping. The distance of the order of ~4 Å corresponded to the literature concerning polyaniline, and allowed hopping to be charged between the chains [24,33]. 

### 3.9. Thermal Properties

**Figure 11:**
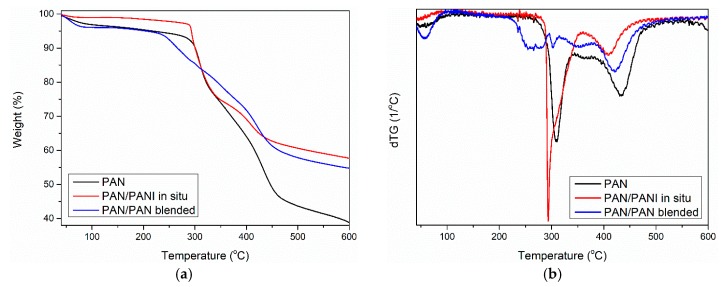
TG (**a**) and DTG (**b**) curves of polymer fibers.

Thermogravimetric analysis of the pure PAN fiber and PANI-containing fibers showed some similarity between the samples (Figure 11). Three mass losses were observed for all materials. The first between 25 and 100 °C (Table 6—T_1_), the second between 200 and 300 °C (Table 6—T_2_), and the third at around 420 °C (Table 6—T_3_). 

**Figure 12:**
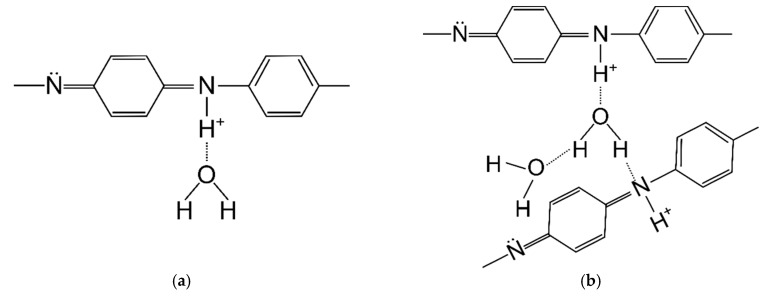
Polyaniline with weak (**a**) and strong (**b**) bound water [30].

The first mass loss was caused by the loss of water bound in the material, which was due to polyaniline adsorbing large amounts of water. Water can be associated with polyaniline macromolecules in two ways: strong and weak. Poorly bound water molecules are connected to the PANI chain with one hydrogen bond (Figure 12a). In contrast, strongly bound water is connected by two hydrogen bonds (Figure 12b), most often between two adjacent PANI chains [25].

Poorly adsorbed water can be removed while drying with nitrogen at room temperature, while the strongly bound one is mostly removed at a temperature in the range of 60–140 °C. Removal of this water is reversible and it can be re-absorbed. However, further removal of water from the polymer at 150–220 °C is an irreversible process and leads to polymer degradation [34,35].

The first derivatives of the TG curve showed that the most moisture was adsorbed in the PAN/PANI in situ fiber. Less water was present in the pure PAN fiber, and the smallest amount was found in the PAN/PANI blended fiber. As pure polyaniline can bind up to 40% by weight of water [36], it is possible to determine its presence in fibers based on the analysis of the water-related peak. An intense peak, much larger than in the reference fiber, was associated with the presence of water in the PAN/PANI in situ fiber. This demonstrated the presence of polyaniline in fibers, which was also confirmed by the FTIR results (Table 5) and XRD (Table 6). A very small amount of adsorbed water in the PAN/PANI blended fiber resulted from the fact that the polyaniline, after the synthesis process, was dried before its dissolution in dimethylformamide.

Further fiber degradation was associated primarily with the phenomenon of polyacrylonitrile cyclization occurring under nitrogen [37]. Analysis of the first derivative of TG curve (i.e., DTG curve) showed that in the case of the pure PAN and PAN/PANI blended samples, the degradation can be described as one-step and starts at around 280 °C. The PAN/PANI blended fiber was characterized by a very rapid decomposition in this temperature range, which persisted up to 350 °C.

The PAN/PANI in situ fiber was decomposed in two stages. It had a wide weight loss of about 10% in the temperature range of 200–290 °C. The initial weight loss in this range was associated with the removal of the rest of the strongly bound water. On the other hand, according to the obtained results and literature data, the weight loss was due to the presence of low-molecular polyaniline fractions in the material, which underwent gradual decomposition [38]. This process is characteristic for PANI degradation, which is why it indicated its presence in the fiber. Another loss beginning at 290 °C and ending at 380 °C was associated with PAN cyclization. Comparing the results obtained in all the fibers showed that the in situ synthesis of polyaniline was a difficult cyclization. This was closely correlated with the FTIR conclusions (Table 5), which indicated electrostatic interactions between the macromolecules of polyacrylonitrile and polyaniline in the PAN/PANI in situ fibers.

The third loss of fiber mass corresponded to the carbonation process of the PAN, which consisted of dehydrogenation and denitrogenation processes [39]. As a result of these processes, a cyclic carbon structure was created. However, when combining the maximum transformation temperature, it can be seen that the lowest value of this parameter was for the PAN/PANI blended fiber. This clearly indicated that the addition of polyaniline introduced by the blended method facilitated the carbonization process.

The total weight loss (Δm_tot_) after measurement was about 15% higher for the pure PAN fiber than for the modified fibers (Table 6). Considering the high thermal resistance of PANI, this proved the effectiveness of both methods of introducing a polymer additive to the fiber. 

**Figure 13:**
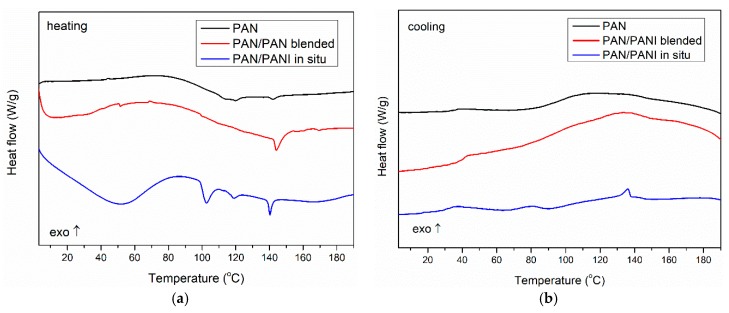
Differential scanning calorimetry (DSC) curve of polymer fibers: (**a**) heating curves; (**b**) cooling curves.

To verify the phase composition of the fibers, DSC measurements were conducted (Figure 13). The obtained results indicated that the pure PAN fiber showed a glass transition at a temperature of about 100 °C both for heating and cooling runs, which was characteristic for polyacrylonitrile [40,41].

In the case of the PAN/PANI in situ fibers’ heating curve, a wide endothermic peak was seen with a maximum at 50 °C, associated with the removal of poorly bound water. An endothermic peak was seen at 102 °C, associated with the desorption of strongly bound water to the PANI [38]. Subsequently, the glass transition in the PAN was observed at about 110 °C, but its exact value was difficult to accurately determine because of the overlap of both processes. At 140 °C, one more endothermic peak was visible, which may be the result of the presence of low molecular weight fractions in the material remaining after the PANI polymerization process (Figure 13a). In the cooling curve, two exothermic peaks were observed, which could be connected with crystallization of previous melted low molecular fractions during heating. Also, a glass transition of PAN at about 100 °C was present (Figure 13b). The obtained results indicated a large heterogeneity of the system, which was also confirmed by previous SEM, mechanical strength, and TG measurements.

In the case of the PAN/PANI blended fibers, only the PAN glass transition (100 °C) was visible in the heating curve, with an additional transition at around 150 °C. Because this temperature was not identical to the glass transition of polyaniline, and was not observed in the front samples (Figure 13a), the authors believe that it was the result of the presence of small amounts of low molecular weight PANI fractions in the material structure, which was well mixed with pure polyacrylonitrile; therefore, the obtained fibers had good mechanical parameters (Table 3) and a smooth structure (Figure 6b). This fraction was homogenized with the rest of the material because during the cooling run, no crystallization peaks were present. Only T_g_ of PAN was observed (Figure 13b). This phenomenon also explains the problem in observing peaks from PANI to XRD, and the presence of only partial peaks in the FTIR.

### 3.10. Electrical Measurements

In order to determine the effect of polyaniline on the electrical properties of the fibers, resistance tests were carried out. The resistance of fibers on the 0.3 cm section was measured using the constant current method, and the results were given as mass-specific resistance Rs (Ωg/cm^2^) (8). This is a parameter used to describe fiber resistance [42]. This measure allows the electrical properties of fibers to be shown in relation to their construction, and not geometric parameters (such as diameter), which are difficult to determine in yarns composed of many fibrils.

(8) $$\mathrm{Rs}=\frac{{\mathrm{RNm}}_{l}}{l\times 10^{5}},$$

where R is the resistance of the sample, N is the number of fibers in the yarn, m_l_ is the linear mass (tex) = (g/km), and l is the length of the sample (cm).

Standard polyacrylonitrile (reference) fibers are dielectrics with a resistance of 10^11^ Ω (Table 7). Both types of fibers doped with polyaniline were tested for resistivity. In the case of the PAN/PANI blended fibers, no change in the fiber resistance was observed, whereas the PAN/PANI in situ fibers were characterized by mass-specific resistances of 5.47 kΩg/cm^2^. These fibers, as shown by the FTIR, XRD, and TG studies, consisted of two phases of different polymers, which were additionally visible in the SEM images. In these fibers, the polyacrylonitrile matrix was responsible for the mechanical properties and thermal stability of the fibers. Polyaniline was responsible for their electrical conductivity. 

In order to compare the obtained results with literature data, it can be assumed that the conductance of the obtained PAN/PANI in situ fibers is of the order 10^−4^ Scm^−1^. This conductance value is lower than that of pure PANI fibers. According to literature data, polyaniline fibers have conductance on the order of 10–100 Scm^−1^ [43,44], but mechanical properties of these fibers are weak. Better mechanical properties, but lower conductance, usually is obtained in composite fibers. Toptaş et al. obtained composite PAN/PANI fibers in a different method that was presented in this paper, but mechanical and electrical properties are comparable [14].

## 4. Conclusions

The article presents two methods of obtaining polyacrylonitrile-polyaniline fibers (PAN/PANI), as well as their physicochemical properties. Polyaniline was used to dope the polyacrylonitrile, which was obtained by synthesis in aqueous solution or dimethylformamide during the preparation of the spinning solution.

It should be noted that in the PAN fiber production process that was doped with PANI, an identical polyacrylonitrile amount of 13.5% was assumed in both the non-doped and PANI-doped fibers, which, as a consequence, influenced the spinning process and physical properties of the obtained fibers. 

During spinning, an attempt was made to maintain identical solution concentrations, spinning bath temperature and water vapor, as well as the pickup and feed speed at the pickup and delivery points. In turn, this was cause for the PAN/PANI in situ fibers to resist stretching in steam, which, in turn, translated into a lowering of their specific strength. During the spinning of the identical solution concentrations, the spinning bath temperature and water vapor, as well as the pickup and feed speed at the pickup and delivery points were maintained. This was cause for the PAN/PANI in situ fibers to resist stretching in steam, which resulted in a lowering of their specific strength.

The fibers obtained by mixing the previously synthesized polyaniline with the polyacrylonitrile fiber spinning solution (PAN/PANI blended) were characterized by:

- blue or green color (depending on pH);
- good mechanical strength;
- smooth and homogeneous surface and cross-section;
- lack of visible peaks from the PANs on the diffraction patterns;
- poor visibility of the peaks—characteristic of PANI on the FTIR spectra;
- one-stage thermal decomposition;
- being dielectric.

Analysis of the above results led to the conclusion that the PANI could be introduced into the fiber in amount and form, which blended well with polyacrylonitrile and formed a homogeneous structure. This additive did not disturb the fiber crystal structure and morphology, but only slightly changed the thermal parameters of the fibers. However, this degree of combination of polymers prevented the transport of electric charges in the fibers; therefore, they remained dielectric. Further research on this modification method will focus on the effective increase of polyaniline content in fibers.

The fibers obtained as a result of the direct synthesis of polyaniline in the spinning solution (PAN/PANI in situ) were characterized by:

- black color;
- poor mechanical strength;
- a heterogeneous and porous surface and cross-section;
- visible peaks from PANI on the diffraction patterns;
- visible peaks characteristic of PANI on the FTIR spectra;
- interactions between the chains of functional groups of the PAN and PANI;
- high water content;
- a multi-stage thermal decomposition;
- good electrical conductivity.

The low mechanical strength of the fibers was mainly due to the lack of fiber stretching, their heterogeneous structure, and low degree of crystallinity. The reduced crystal crystallinity in comparison to the PAN fibers resulted from the stiffness of the structure caused by the presence of interactions between the PAN and PANI chains visible on the FTIR spectra. These fibers exhibited electro-conductive properties at the level of 5.47 kΩg/cm^2^, which can be used to construct electrical connections in textronic clothing. Moreover, thanks to the reversible color change, which depends on the pH of the environment in which they are found, they can also be used as pH sensors in protective clothing. In further work, the authors will develop research to obtain electro-conducting PAN/PANI fibers with the highest possible mechanical strength. For this purpose, the study will focus primarily on the modification of parameters for obtaining a spinning solution, as well as the spinning itself.

## Figures and Tables

**Table 1:** The value of flow behavior index n and the flow consistency index k of the spinning solutions.

Spinning Solution	n	k (Pa·s^n^)
PAN	0.85	32.84
PAN/PANI blended	0.89	19.97
PAN/PANI in situ	0.87	24.03

**Table 2:** Description of characteristic vibrations observed in the FTIR spectra of the tested substance.

Band Origin ^1^	PAN	PANI	PAN/PANI Blended	PAN/PANI In Situ
υ N–H	3480	-	3445	3440
υ C–H in CH_2_	2940	-	2926	2940
υ C≡N	2243	-	2243	2244
υ C=O	1733	-	1731	1726
υ C=C	1629	-	1615	1629
υ C=N in Q	-	1576	1588	-
δ C–H in CH_2_	1454	-	1453	1445
υ C–N^+^	-	1414	-	1414
υ C–N in Q and B	-	1302	1306	1300
δ C–H in CH	1227	-	1250	1260
υ Q=N–B and B–NH–B (deprotonated PANI)	-	-	1160	1194
υ Q=N^+^H–B and B–N^+^H–B (protonated PANI)	-	1144	-	-
δ C–H in 1,2,4 trisubstituted B and Q	-	832	804	879

^1^ Where: υ—stretching, δ—bending, B—benzene ring, and Q—quinoid ring.

**Table 3:** Mechanical parameters of the fibers.

Sample	m_l_ (tex)	E (cN/tex)	F_brk_ (cN)	F_max_ (cN)	WtP (cN/tex)	ε (%)	λ (mm)
PAN	95.59	583.49	1983.35	3680.89	38.51	18.59	9.30
PAN/PANI blended	100.03	472.59	847.48	2011.36	18.39	8.73	4.37
PAN/PANI in situ	233.7	164.85	275.89	1465.97	6.27	11.27	5.64

where: m_l_—linear mass; E—Young’s modulus; Fbrk—average breaking force of fiber multifilament; Fmax—maximum breaking force of fiber multifilament; WtP—specific strength of fiber multifilament; ε—average relative elongation; and λ—average absolute elongation.

**Table 4:** The degree of fiber crystallinity.

Sample	The Degree of Fiber Crystallinity
PAN	0.53
PAN/PANI blended	0.53
PAN/PANI in situ	0.32

**Table 5:** Crystallinity properties of fibers.

Sample	hkl	Polymer	Diffraction Peak (^o^)	L (nm)	d-Spacing (Å)	R (Å)	ε (-)
PAN reference	(001)	PAN	16.85	5.7	3.95	6.58	0.00092
PAN/PANI blended		PAN	16.80	6.0	3.96	6.60	0.00086
PAN/PANI in situ		PAN	16.77	5.7	3.97	6.61	0.00091
	(111)	PANI	26.19	15.8	3.40	4.26	0.00053
	(005)	PANI	23.42	17.9	3.80	4.75	0.00041

**Table 6:** Data obtained from the thermogravimetric curve, T_1,2,3_—temperature of subsequent losses in mass.

Sample	Onset (°C)	T_1_ (°C)	T_2_ (°C)	T_3_ (°C)	Δm_tot_ (%)	m_PANI_ (%)
PAN reference	296	-	308	434	61.22	-
PAN/PANI blended	290	-	294	409	42.32	18.9
PAN/PANI in situ	231	265	303	421	45.26	16.0

**Table 7:** Electrical properties of obtained fiber.

Sample	Mass-Specific Resistance	Units
PAN reference	~10^11^	Ωg/cm^2^
PAN/PANI blended	~10^11^	Ωg/cm^2^
PAN/PANI in situ	5470	Ωg/cm^2^
